# Marine-Derived Sterols from *Saccharina japonica*: Potential Antibacterial Activity and Target Prediction Against Bacterial Pathogens Through Integrated In Vitro and In Silico Approaches

**DOI:** 10.3390/pathogens15060576

**Published:** 2026-05-27

**Authors:** Eun-Seop Lee, Chae-Min Kim, Si-Heon Song, Su-Bin Jeon, Byeong-Su Kang, Md Sekendar Ali, Eon-Bee Lee

**Affiliations:** 1 Department of Aquatic Life Medicine, Pukyong National University, Busan 48513, Republic of Korea; 2 Department of Pharmacy, International Islamic University Chittagong, Kumira, Chittagong 4318, Bangladesh

**Keywords:** seaweed, antibacterial activity, kinetic analysis, drug-likeness, ligands

## Abstract

Marine natural products, including seaweeds, sponges, and marine microorganisms, have emerged as promising sources of bioactive compounds with diverse pharmacological properties. We investigated the antibacterial and antioxidant potential of ethanol extracts (30%, 60%, and 90%) from *Saccharina japonica* collected from two Korean coastal regions, Gijang and Wando, and evaluated their bioactive metabolites through integrated in vitro and in silico approaches. Among the extracts, the 60% ethanol fraction exhibited the highest total phenolic content and strongest 2,2-diphenyl-1-picrylhydrazyl (DPPH) radical-scavenging activity, indicating superior antioxidant capacity. Antibacterial assays revealed selective concentration-dependent inhibition against *Staphylococcus aureus*, while limited effects were observed against *Escherichia coli*. Kinetic analysis further demonstrated growth suppression of *S. aureus* at higher concentrations. Molecular docking was performed against multiple bacterial proteins, including DNA gyrase, topoisomerase IV, and tyrosyl-tRNA synthetase. Sterol compounds, particularly fucosterol and 24-methylene cholesterol, showed strong binding affinities across key targets, suggesting their potential role as multitarget antibacterial agents. ADMET predictions indicated favorable pharmacokinetic properties, although high lipophilicity and potential hERG II inhibition were noted. Overall, while the antibacterial effects observed were moderate, these findings suggest that marine-derived sterols from *S. japonica* may represent compounds of interest for further mechanistic investigation and optimization in complementary antibacterial strategies.

## 1. Introduction

The widespread and rapid use of antibiotics, together with inappropriate prescription and misuse, has accelerated the emergence of resistant bacterial strains. Antimicrobial resistance has reduced the effectiveness of conventional antibiotics, thereby increasing the need for alternative antimicrobial agents. Therefore, identifying alternative sources of bioactive compounds with chemically diverse scaffolds has become an important complementary strategy to address the growing challenge of antimicrobial resistance [1,2]. In this context, natural bioactive compounds derived from marine organisms have attracted increasing attention as potential alternatives with antimicrobial and antioxidant properties. Among these, seaweeds—valued for their biodiversity, productivity, and accessibility—harbor diverse bioactive compounds such as phenolics, terpenoids, flavonoids, alkaloids, and polysaccharides, some with unique structures that exhibit antioxidant, anti-inflammatory [3], anticancer, antidiabetic, and antimicrobial activities [4]. Although previous studies have reported the antioxidant and antimicrobial activities of seaweed-derived compounds, fewer studies have examined how extraction solvent concentration and geographical origin jointly influence the bioactivity, metabolite profiles, and predicted pharmacokinetic properties of *S. japonica* extracts. *Saccharina japonica* (syn. *Laminaria japonica*), a large brown alga (Phaeophyceae) widely distributed in Northeast Asian coastal regions and long used as a traditional food and medicinal resource, contains diverse bioactive compounds with antimicrobial and antioxidant activities [5]. The bioactivity of natural products can vary depending on the polarity and concentration of the extraction solvent. Previous studies have demonstrated that the chemical composition of ethanol extracts from brown algae varies according to ethanol concentration [6]. Furthermore, geographical factors such as water temperature, salinity, and nutrient availability can influence the biochemical composition of seaweeds, leading to differences in biological activity [7]. Therefore, in this study, *S. japonica* samples collected from two Korean coastal regions, Gijang and Wando, were compared to explore whether the regional origin is associated with differences in antimicrobial and antioxidant potential.

However, studies that simultaneously compare the antimicrobial and antioxidant potential of *S. japonica* extracts according to coastal origin and extraction conditions remain limited [8]. In particular, little is known about how ethanol concentration and geographical origin jointly influence fucoxanthin content, antibacterial selectivity, antioxidant capacity, and predicted drug-likeness of *S. japonica*-derived compounds. Accordingly, ethanol extracts of *S. japonica* obtained from two different coastal regions were prepared using 30%, 60%, and 90% ethanol, and their biological activities were comprehensively evaluated. These included antimicrobial effects against two representative foodborne pathogens (*Escherichia coli* and *Staphylococcus aureus*) and antioxidant potential assessed through total phenolic content (TPC) and DPPH radical scavenging assays. To bridge experimental findings with drug discovery, we performed molecular docking and absorption, distribution, metabolism, excretion, and toxicity (ADMET) predictions using SwissADME and pkCSM, providing biological validation together with pharmacokinetic and toxicity insights into the applicability of *S. japonica*-derived compounds as novel antimicrobial agents.

## 2. Materials and Methods

### 2.1. Bacterial Strains and Growth Medium

The bacterial strains used in this study, *E. coli* ATCC 35218 and *S. aureus* ATCC 29213, were obtained from American Type Culture Collection (ATCC, Manassas, VA, USA). The strains were preserved at −80 °C using a bead-based cryopreservation system. Before each experiment, frozen stocks were streaked onto Mueller–Hinton agar (MHA) plates (KisanBio, Seoul, Republic of Korea) and incubated at 37 °C for 18 to 24 h. A single colony was transferred to Mueller–Hinton broth (MHB) and cultured at 37 °C for 18 to 24 h to obtain an actively growing culture, then subcultured under the same conditions before use in antimicrobial assays.

### 2.2. Preparation of S. japonica Material

The dried fronds of *S. japonica* (syn. *L. japonica*) were purchased from Herbmaeul Co., Ltd., Cheongju, Republic of Korea, a commercial supplier providing authenticated edible kelp products sourced from Gijiang (35.2392° N, 129.1580° E) and Wando (32.8302° N, 79.9510° W), South Korea. The raw material consisted of 100% dried kelp leaves, with no preservatives added and stored under sealed cool conditions (1–15 °C). Detailed information included the supplier’s documentation (INCI: *Laminaria japonica*, CAS No. 223751-72-2/92128-82-0), processing (selected and dried), and packaging (HDPE mixed film; 1 kg unit). The dried seaweed was ground using a blender (DMW-3500, Daewon Co., Ansan, Republic of Korea) and stored at −20 °C until use.

### 2.3. Preparation of S. japonica Extracts

The extracts of *S. japonica* were prepared with slight modifications to the method described by Cai et al. [9]. A total of 10 g of ground *S. japonica* samples collected from Gijang and Wando were extracted with 30%, 60%, or 90% ethanol (EtOH) at a solvent-to-solid ratio of 1:25 (*w*/*v*). The extraction was carried out at 80 °C for 12 h. The extracts were filtered through Whatman No. 2 filter paper, and the filtrates were concentrated under reduced pressure at 45 °C using a rotary evaporator (RE 212-D, Yamato Co., Tokyo, Japan) to remove the solvent. The concentrates were mixed with a small amount of ethanol and 25 mL of distilled water, followed by ultrasonication to obtain the final concentrated extracts. Each sample was extracted twice under the same conditions, and the resulting extracts were pooled before concentration and freeze drying. The pooled extracts were stored at −80 °C and subsequently freeze-dried to obtain the final ethanol extract powders. The ethanol extract of *S. japonica* revealed an average yield of 5.23%. For subsequent biological assays, the freeze-dried extract powders were dissolved in 30% EtOH to prepare 80 mg/mL stock solutions and diluted to the assay-specific working concentrations.

### 2.4. Determination of Fucoxanthin Content by HPLC

Fucoxanthin was selected as the target compound for HPLC analysis because it is a representative carotenoid of brown seaweeds, including *S. japonica*, and has been reported to exhibit antioxidant and antibacterial properties. Fucoxanthin in the *S. japonica* ethanol extract was quantified using an HPLC system (Agilent 1200 series) equipped with a UV–Vis detector and an Eclipse XDB-C18 column. The analysis was conducted under slightly modified conditions based on the method of Kanazawa et al. [10]. The mobile phase consisted of isocratic ACN: water (70:30, *v*/*v*) at a flow rate of 1.0 mL/min, with the column maintained at 35 °C and detection at 449 nm. Fucoxanthin standards (0.5–20 μg/mL) were prepared from a 0.1 mg/mL stock solution, and the calibration curve was constructed over the range of 1–20 μg/mL. Method validation was performed using fucoxanthin standard solutions by evaluating linearity, limit of detection (LOD), limit of quantification (LOQ), and precision. Linearity was assessed using standard calibration curves, and precision was expressed as relative standard deviation (RSD). Sample extracts were prepared by dissolving freeze-dried powder in 70% ACN, followed by sonication, centrifugation, and filtration through a 0.45 μm syringe filter prior to HPLC injection.

### 2.5. In Vitro Studies

#### 2.5.1. Antioxidant Activity

##### Determination of Total Phenolic Content (TPC)

The total phenolic content (TPC) was determined using the Folin–Ciocalteu method [11]. The extracts were reacted with Folin–Ciocalteu reagent and sodium carbonate, and the absorbance was measured at 725 nm. Gallic acid (GC) was used to generate the calibration curve. All measurements were performed in triplicate, and TPC was expressed as μg GC equivalents (GAE) per gram of dried sample.

##### DPPH Radical Scavenging Activity

The DPPH radical scavenging activity was assessed according to the method of Wang et al. [11], with minor modifications. Briefly, sample solutions prepared in 50% ethanol were mixed with 0.1 mM DPPH and incubated in the dark, and the absorbance was measured at 517 nm. Background absorbance from the sample color was corrected using corresponding blanks. A control was prepared without a sample. The DPPH radical-scavenging activity (%) was calculated using the following equation [12]:

$$Scavenging\;effect\;(\%)\;=\;(1-\frac{A_{sample+DPPH}-A_{sample}}{A_{control}})\times 100$$

##### Growth Kinetics of Bacterial Strains

To quantitatively assess the time-dependent growth patterns of *E. coli* and *S. aureus*, a colony-forming unit (CFU)-based growth curve assay was conducted. The initial inoculum was adjusted to 5 × 10^5^ CFU/mL and cultured in MHB at 37 °C for 24 h. Bacterial growth was evaluated at 11 time points: 0, 0.5, 1, 1.5, 2, 2.5, 3, 4, 6, 12, and 24 h. At each time point, CFUs were determined using the standard plate count method. The resulting data were converted to a log_10_ scale to generate growth curves. The experiment was performed in triplicate under identical conditions to ensure reproducibility.

#### 2.5.2. Antibacterial Activity

##### Minimum Inhibitory Concentration (MIC)

The MIC was determined against *E. coli* and *S. aureus* to quantitatively evaluate the antibacterial activity of *S. japonica* extracts. The MIC test was performed using the broth dilution method in accordance with the Clinical and Laboratory Standards Institute (CLSI) guidelines [13]. Six types of freeze-dried extracts were prepared at an initial concentration of 20 mg/mL. Each extract was added to MHB, and bacterial suspensions were inoculated to reach a final concentration of 5 × 10^5^ CFU/mL. The cultures were incubated at 37 °C for 24 h. Gentamicin was included as a reference antibiotic control and tested under the same experimental conditions. The cultures were incubated at 37 °C for 24 h. MIC was defined as the lowest concentration of *S. japonica* extract that inhibited visible bacterial growth.

All extracts were dissolved in 30% ethanol. To evaluate the antimicrobial effect of the solvent itself, solvent control was prepared by adding the same volume of 30% ethanol (without extract) to MHB, under conditions identical to those of the test groups. All experiments were conducted in triplicate to ensure reproducibility.

##### Kinetic Analysis of Bacterial Growth Inhibition

Kinetic analysis of bacterial growth inhibition was performed using the *S. japonica* extract that exhibited a relatively low MIC value to evaluate its time-dependent effect on bacterial growth. The test groups were treated with the extract at concentrations of 1 × MIC, 2 × MIC, and 4 × MIC. A growth control group was maintained without extract treatment. The initial bacterial inoculum was adjusted to 5 × 10^5^ CFU/mL in MHB, and the cultures were incubated at 37 °C for 24 h. Bacterial growth was monitored by measuring the optical density at 600 nm (OD_600_) using a microplate reader (Epoch 2, Agilent BioTek, Santa Clara, CA, USA). OD_600_ values were recorded at regular intervals to assess the time-dependent growth inhibition over time, and the data were used to construct growth inhibition curves.

### 2.6. In Silico Studies

#### 2.6.1. Protein Preparation

The crystal structures of the target proteins were obtained from the RCSB Protein Data Bank (PDB). The selected targets included proteins involved in essential bacterial processes, such as DNA topology regulation, cell wall biosynthesis, and protein synthesis, as well as *S. aureus* virulence-related proteins. Specifically, DNA gyrase B (PDB IDs: 1KZN, 5L3J, and 3TTZ), topoisomerase IV (PDB ID: 3FV5), MurA (PDB ID: 1UAE), FemA (PDB ID: 1LRZ), and TyrRS (PDB ID: 1JIJ) were included as antibacterial targets associated with bacterial survival and proliferation. In addition, toxic shock syndrome toxin-1 (TSST-1; PDB ID: 2QIL) and C(30) carotenoid dehydrosqualene synthase CrtM (PDB ID: 2ZCO) were included as *S. aureus* virulence-related targets rather than direct growth inhibition targets. All protein structures were preprocessed and prepared using the DockPrep module of UCSF ChimeraX.

#### 2.6.2. Ligand Preparation

The metabolite profile of *S. japonica* was obtained from the KNApSAcK species–metabolite database. The three-dimensional structures of the selected ligands were retrieved in SDF format from the PubChem database. Structural optimization of each compound was performed using Avogadro software (version 1.2.0). The steepest descent method was applied in combination with MMFF94 (Merck Molecular Force Field), a molecular mechanics-based force field.

Furthermore, the most stable conformer of each ligand was identified by applying the “scoring function energy” module within Avogadro, and this lowest-energy conformer was used consistently for all subsequent analyses. Finally, each optimized compound was imported into PyRx software (version 0.8) and converted into PDBQT format using the “Load Molecule” function [14].

#### 2.6.3. Molecular Docking Analysis

Molecular docking was performed for 18 compounds (Table S1) derived from *S. japonica* using the AutoDock Vina (version 1.1.1) engine within the PyRx virtual screening tool [15]. The three-dimensional structures of the target proteins were imported into PyRx and converted into PDBQT format, including polar hydrogens. Ligand structures were energy minimized and converted into PDBQT format, with all rotatable bonds treated as flexible.

Grid boxes were defined around the native ligand binding sites or previously reported active site residues. Detailed docking sites, grid center coordinates, grid box parameters, and redocking RMSD values are summarized in Table S2. For proteins containing a co-crystallized ligand, docking validation was performed by redocking the native ligand into its original binding pocket. RMSD values below 2.0 Å were considered acceptable for reproducing the experimental binding pose. For proteins without a co-crystallized ligand, redocking validation was not applicable, and the docking site was defined based on active site residues reported in previous studies [16,17,18].

The binding affinity (affinity value) of each complex was determined based on the best docking pose with the lowest binding energy. The binding interactions of the docked complexes, including hydrogen bonding patterns and bond lengths, were analyzed using Discovery Studio Visualizer.

#### 2.6.4. Drug-Likeness and ADMET Profile

Compounds derived from *S. japonica* that were included in the molecular docking analysis were further evaluated for drug-likeness and ADMET-related properties. Drug-likeness, physicochemical properties, pharmacokinetics, and oral bioavailability were assessed using the SwissADME web tool [19]. In addition, the pkCSM server was employed to predict the pharmacological profiles of the selected compounds, including toxicity, efficacy, and distribution characteristics [20].

## 3. Results and Discussion

### 3.1. Quantification of Fucoxanthin by HPLC

**Figure 1:**
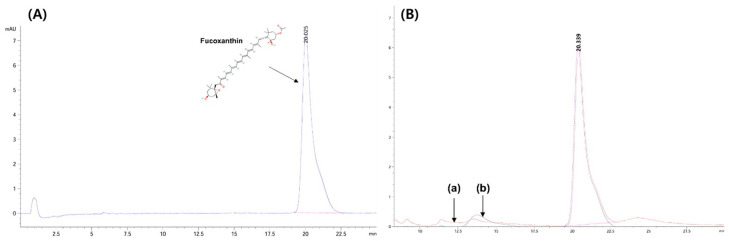
HPLCchromatograms of fucoxanthin and SJE. (**A**) Fucoxanthin standard showing a characteristic peak at its retention time. (**B**) HPLC chromatograms of *S. japonica* ethanol extracts from two regions: KSJE (a, red line) and WSJE (b, blue line).

HPLC analysis confirmed the presence of fucoxanthin in the ethanol extracts of *S. japonica* collected from Gijang and Wando (Figure 1). The fucoxanthin standard showed a distinct peak at 20.025 min, and both extracts exhibited matching retention times, indicating successful identification of the compound. The calibration curve showed good linearity over the concentration range of 1–20 µg/mL, with an R^2^ value of 0.994. The LOD and LOQ were estimated to be 0.5 µg/mL and 1 µg/mL, respectively. The peak intensity was higher in the WLJE extract, and quantitative analysis revealed fucoxanthin levels of 3.57 ppm in WLJE and 2.56 ppm in KLJE, indicating a clear regional difference in fucoxanthin content. These concentrations were calculated from the fucoxanthin standard calibration curve, and HPLC injection repeatability showed an RSD of 3.58%. The detection of fucoxanthin in both extracts is consistent with previous research reporting fucoxanthin as a major carotenoid in brown seaweeds, particularly *S. japonica*. Fucoxanthin is known to exhibit diverse biological functions, including antioxidant, antibacterial, anti-inflammatory, and cytoprotective effects [21,22]. Therefore, its presence in the extracts suggests that both GLJE and WLJE may retain these inherent bioactivities. These results demonstrate that *S. japonica* ethanol extracts from both regions contain fucoxanthin, supporting their potential use as functional materials in antioxidant or antimicrobial evaluations.

### 3.2. Antioxidant Activity

**Figure 2:**
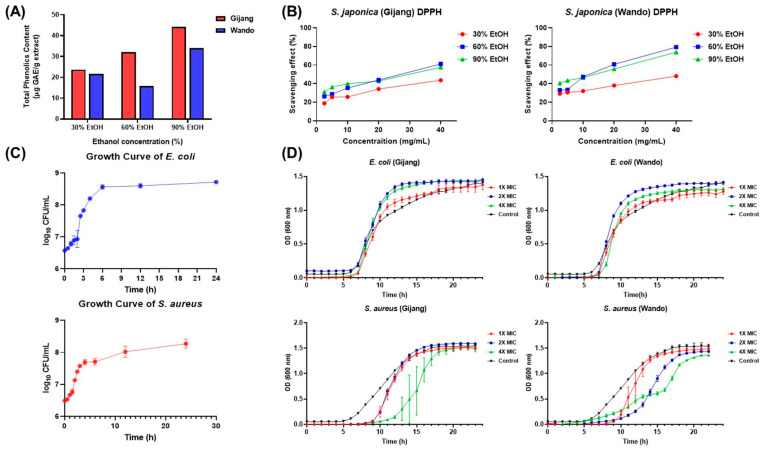
Antioxidant activity and antibacterial assays of *S. japonica* extracts. (**A**) TPC of *S. japonica* extracts. (**B**) DPPH radical scavenging activity. (**C**) Growth curves of *E. coli* and *S. aureus*. The blue and red lines represent the growth curves of *E. coli* and *S. aureus*, respectively. (**D**) Kinetic analysis of bacterial growth inhibition.

The total phenolic content (TPC) and DPPH radical scavenging activity of *S. japonica* extracts were analyzed to evaluate antioxidant potential (Figure 2A). TPC values were calculated from the gallic acid calibration curve, and DPPH activity was expressed as percentage radical-scavenging activity after blank correction and normalization to the control. TPC varied depending on solvent polarity and geographical origin. Gijang-derived extracts showed a steady increase with ethanol concentration, with G90% exhibiting the highest value (44.24 μg GAE/g), followed by G60% (32.09 μg GAE/g) and G30% (23.74 μg GAE/g). In contrast, Wando-derived samples did not follow a clear trend; W90% displayed a relatively high TPC (34.05 μg GAE/g), while W60% and W30% yielded 15.74 and 21.59 μg GAE/g, respectively. These results indicate that both solvent type and geographical origin significantly affect the phenolic content, which is closely correlated with antioxidant capacity [23]. The higher TPC values in Gijang samples suggest environmental or seasonal variations influencing metabolite accumulation, consistent with previous findings in brown algae [7].

DPPH radical scavenging activity further supported these results (Figure 2B). All extracts showed a concentration-dependent increase, with maximal activity at 40 mg/mL. The G60% extract exhibited the strongest activity (61.17%), followed by G90% (57.62%) and G30% (43.63%), while among Wando extracts, W60% showed the highest value (79.29%), followed by W90% (73.88%) and W30% (48.07%). The pronounced antioxidant capacity of the 60% extracts aligns with prior reports that intermediate ethanol concentrations (50–60%) yield the most efficient extraction of antioxidant compounds [24]. Phenolic compounds in brown algae may have been associated with diverse bioactivities, including antioxidant and antimicrobial properties [25]. Although antioxidant activity does not directly establish antibacterial efficacy, it provides complementary information on the phenolic-associated bioactive potential of *S. japonica* extracts and helps evaluate their possible use as multifunctional bioactive materials.

Collectively, these findings highlight the importance of solvent polarity and regional conditions in optimizing the extraction of phenolic compounds, suggesting that *S. japonica* serves as a valuable source of antioxidant bioactives for pharmaceutical, nutraceutical, and cosmetic applications.

### 3.3. Growth Kinetics of Bacterial Strains

*E. coli* exhibited rapid growth within the first 6 h, reaching approximately 5 × 10^8^ CFU/mL, after which the population remained stable until 24 h. In contrast, *S. aureus* showed a rapid increase in cell density between 4 and 6 h, reaching a comparable density at around 10 h, and subsequently maintained a stationary phase until 24 h. CFU values were converted to log10 CFU/mL before constructing the growth curves. These growth kinetics are presented in Figure 2C.

### 3.4. Antibacterial Activity

The antibacterial activity of *S. japonica* ethanol extracts exhibited species-dependent patterns. Against *E. coli*, all extracts—including 30% ethanol controls—showed negligible inhibition, with uniform MIC values of 10 mg/mL. In contrast, *S. aureus* displayed higher sensitivity; G60% and W60% extracts recorded the lowest MICs (2.5 mg/mL and 1.25 mg/mL), while 90% extracts (G90%, W90%) showed MICs of 5 mg/mL (Table 1). Importantly, the MIC values of *S. japonica* extracts were relatively high compared with those typically reported for conventional antibiotics, indicating limited antibacterial potency. Although the extracts showed partial inhibitory activity, particularly against *S. aureus*, their antibacterial effect should be interpreted cautiously. These findings suggest that *S. japonica* extracts may be more appropriate as supplementary bioactive components in multifunctional formulations rather than as primary antibacterial agents.

Kinetic analysis of bacterial growth inhibition further supported these results (Figure 2D). *E. coli* growth was not inhibited within 24 h, and in some cases, treated groups surpassed control levels after 8 h. However, *S. aureus* growth was markedly suppressed at higher concentrations (4 × MIC), with Wando extracts inhibiting growth for approximately 15 h and Gijang extracts for about 10 h. Because this assay was based on OD_600_ measurements, these results indicate time-dependent growth suppression rather than confirmed bactericidal activity.

These species-specific differences can be attributed to bacterial cell wall structures: the outer membrane of Gram-negative *E. coli*—rich in lipopolysaccharides (LPS)—acts as a barrier to antimicrobial penetration, whereas Gram-positive *S. aureus* lacks this structure, allowing direct interaction with active compounds [26,27]. Similar selectivity has been reported in brown algae extracts, which generally show stronger inhibition against Gram-positive bacteria [28]. However, because this study evaluated antibacterial activity using only two representative bacterial strains, *E. coli* and *S. aureus*, the antibacterial spectrum of *S. japonica* extracts may not be fully generalized from the present results. Further studies using a broader range of Gram-positive and Gram-negative bacterial strains are therefore needed. Considering these, *S. japonica* extracts may not serve as potent standalone antibacterial agents; nevertheless, their partial inhibitory and antioxidant effects suggest potential use as supplementary bioactive components in multifunctional formulations.

### 3.5. Molecular Docking

**Figure 3:**
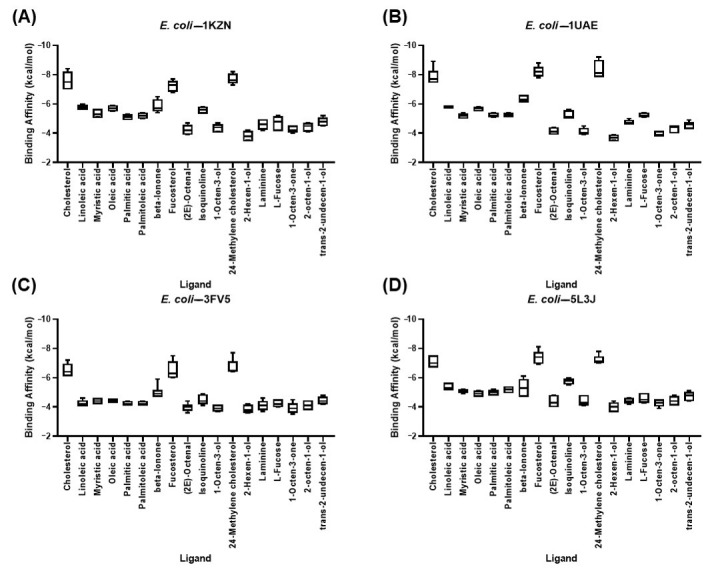
Binding affinity analysis between *S. japonica*-derived ligands and *E. coli* target proteins. (**A**) 1KZN; (**B**) 1UAE; (**C**) 3FV5; (**D**) 5L3J.

**Figure 4:**
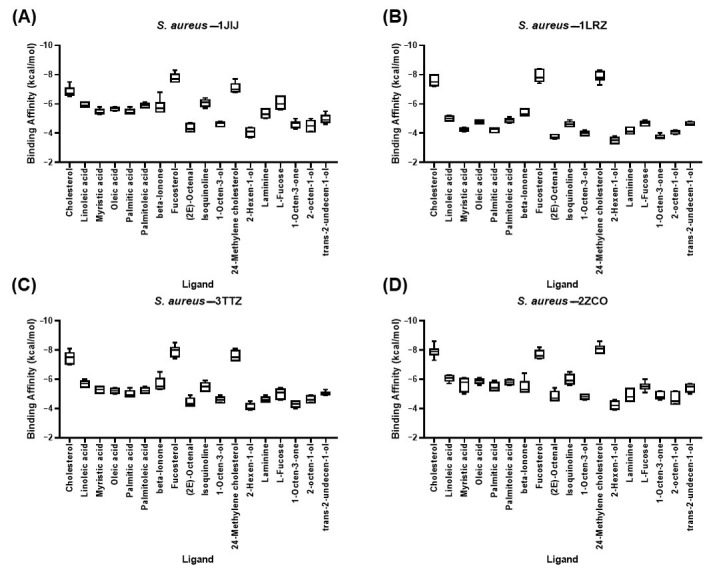
Binding affinity analysis between *S. japonica*-derived ligands and *S. aureus* target proteins. (**A**) 1JIJ; (**B**) 1LRZ; (**C**) 3TTZ; (**D**) 2ZCO.

**Figure 5:**
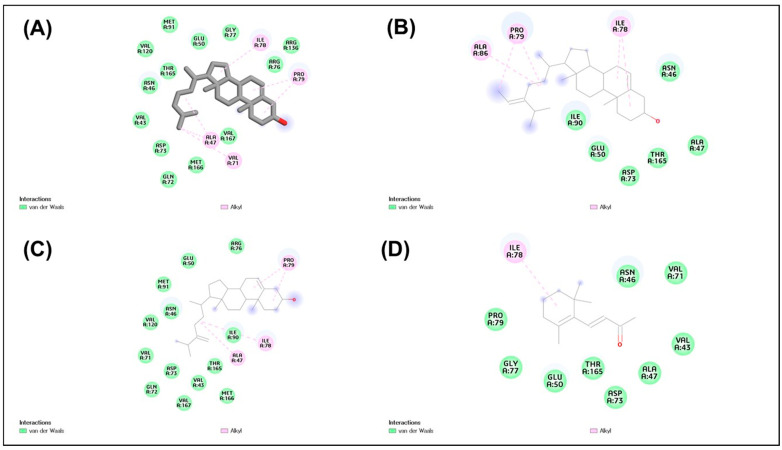
Binding interactions between an *L. japonica*-derived compound and *E. coli* protein 1KZN. (**A**) Cholesterol; (**B**) fucosterol; (**C**) 24-methylene cholesterol; (**D**) β-ionone.

**Figure 6:**
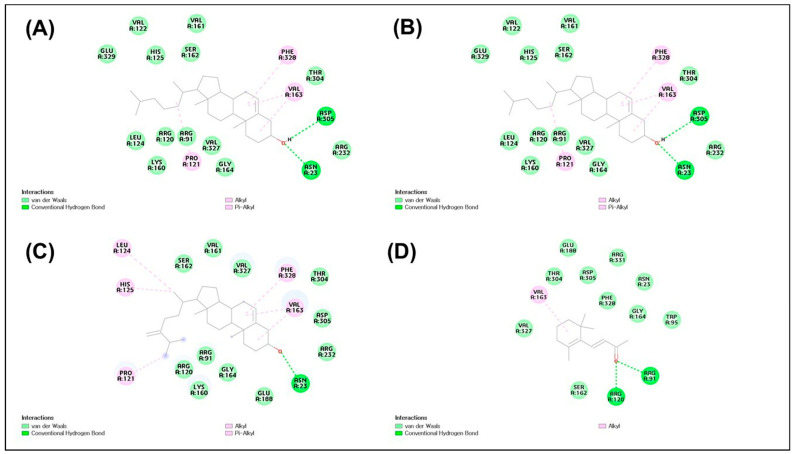
Binding interactions between an *L. japonica*-derived compound and *E. coli* protein 1UAE. (**A**) Cholesterol; (**B**) fucosterol; (**C**) 24-methylene cholesterol; (**D**) β-ionone.

**Figure 7:**
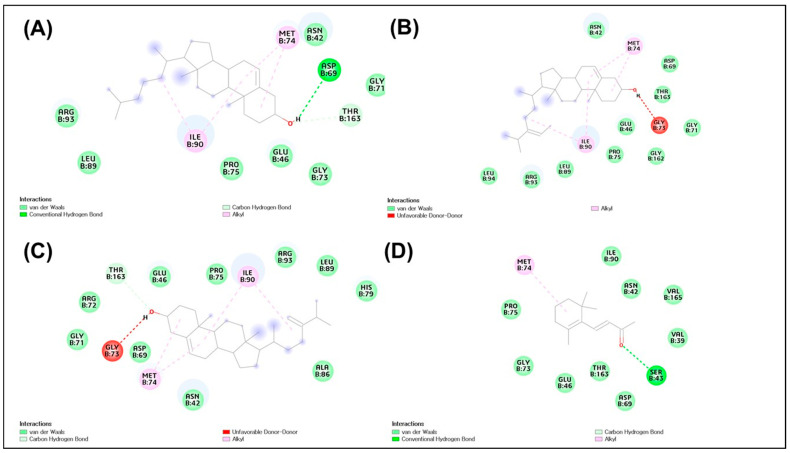
Binding interactions between an *L. japonica*-derived compound and *E. coli* protein 3FV5. (**A**) Cholesterol; (**B**) fucosterol; (**C**) 24-methylene cholesterol; (**D**) β-ionone.

**Figure 8:**
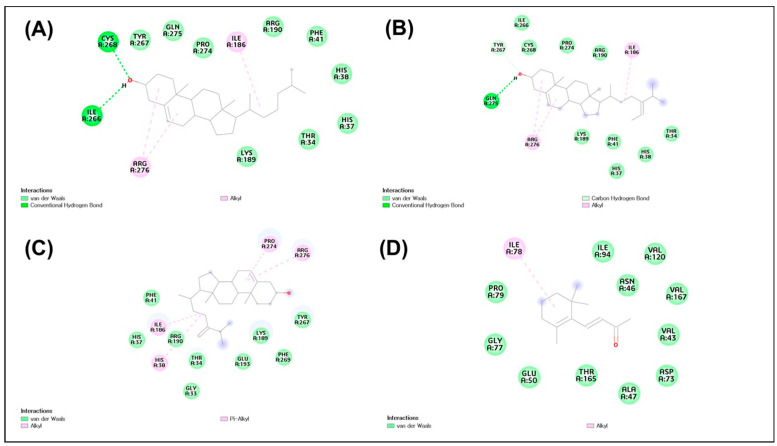
Binding interactions between an *L. japonica*-derived compound and *E. coli* protein 5L3J. (**A**) Cholesterol; (**B**) fucosterol; (**C**) 24-methylene cholesterol; (**D**) β-ionone.

**Figure 9:**
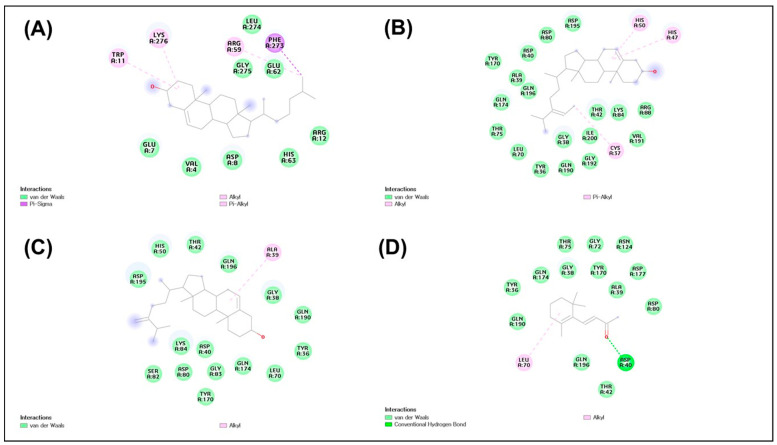
Binding interactions between an *L. japonica*-derived compound and *S. aureus* protein 1JIJ. (**A**) Cholesterol; (**B**) fucosterol; (**C**) 24-methylene cholesterol; (**D**) β-ionone.

**Figure 10:**
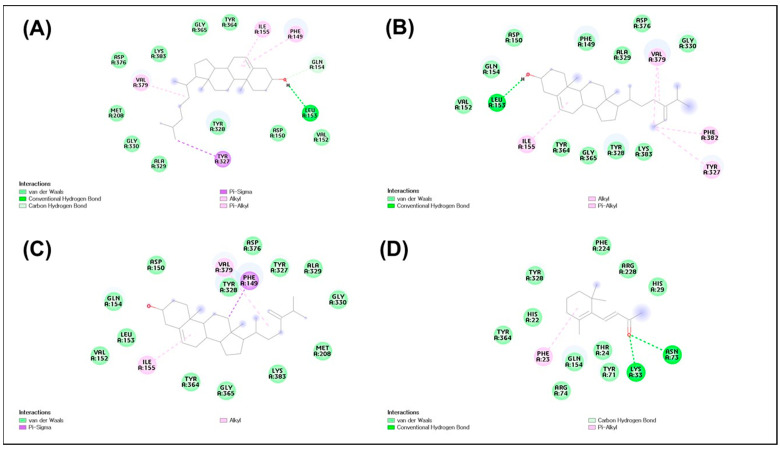
Binding interactions between an *L. japonica*-derived compound and *S. aureus* protein 1LRZ. (**A**) Cholesterol; (**B**) fucosterol; (**C**) 24-methylene cholesterol; (**D**) β-ionone.

**Figure 11:**
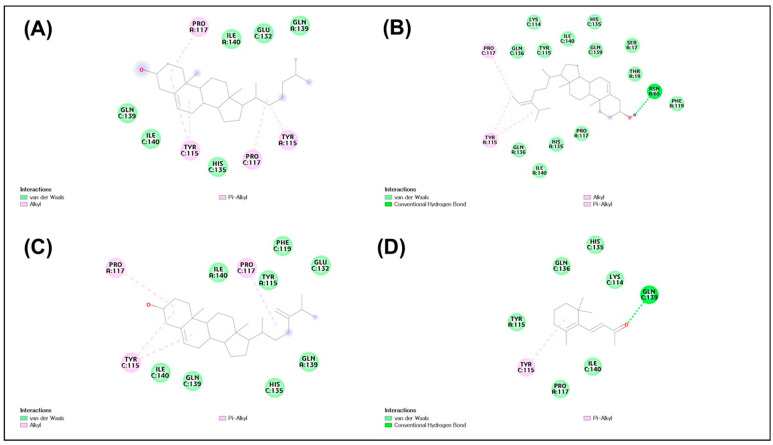
Binding interactions between an *L. japonica*-derived compound and *S. aureus* protein 2QIL. (**A**) Cholesterol; (**B**) fucosterol; (**C**) 24-methylene cholesterol; (**D**) β-ionone.

**Figure 12:**
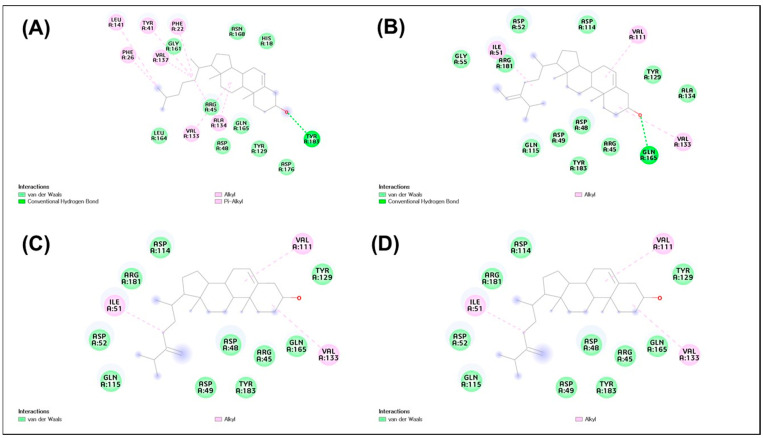
Binding interactions between an *L. japonica*-derived compound and *S. aureus* protein 2ZCO. (**A**) Cholesterol; (**B**) fucosterol; (**C**) 24-methylene cholesterol; (**D**) β-ionone.

**Figure 13:**
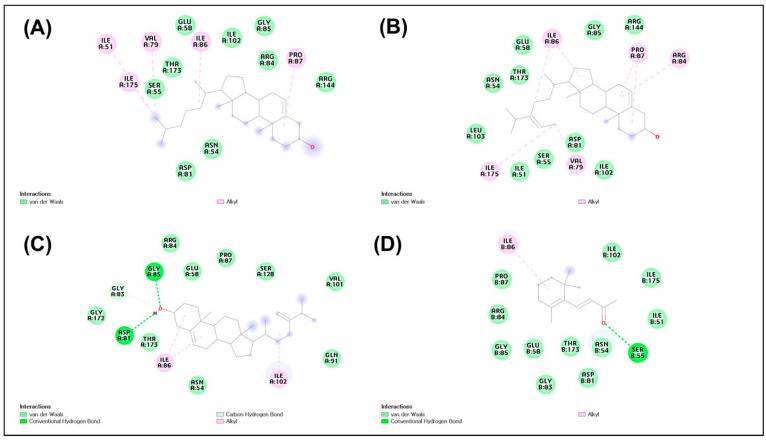
Binding interactions between an *L. japonica*-derived compound and *S. aureus* protein 3TTZ. (**A**) Cholesterol; (**B**) fucosterol; (**C**) 24-methylene cholesterol; (**D**) β-ionone.

Molecular docking simulations showed that most compounds exhibited binding energies below −4 kcal/mol, suggesting potential interactions with the selected bacterial targets, although these values should be interpreted as weak to modest predicted binding rather than strong biological relevance (Figure 3 and Figure 4). Sterol derivatives—particularly fucosterol, 24-methylene cholesterol, and cholesterol—showed relatively favorable docking scores across all eight target proteins, forming stable hydrophobic and hydrogen-bond interactions within active sites (Figure 5, Figure 6, Figure 7, Figure 8, Figure 9, Figure 10, Figure 11, Figure 12 and Figure 13). Fucosterol achieved the highest docking scores, consistent with previous studies reporting its broad-spectrum antimicrobial activity [29].

β-Ionone also demonstrated binding affinities ranging from −5.9 to −6.8 kcal/mol, aligning with the literature describing the strong inhibition of *S. aureus* and *Bacillus subtilis* [30]. Its known involvement in the biosynthesis of chalcones—flavonoid precursors with antibacterial, anticancer, and anti-inflammatory effects—supports the potential for multitarget or synergistic mechanisms [31]. In contrast, D-galactose and isoquinoline displayed selective binding patterns, indicating possible target-specific actions [32]. Overall, sterol derivatives such as fucosterol may represent compounds of interest related to the antimicrobial potential of *S. japonica*. However, because the in vitro assays were performed using crude ethanol extracts whereas molecular docking was conducted with selected individual compounds, the docking results may not provide a direct explanation of the extract-level bioactivity. Rather, they provide preliminary insight into possible interactions between *S. japonica*-derived metabolites and bacterial targets. Further validation using fractionated extracts or purified compounds is needed to clarify the contribution of individual compounds to the observed biological activities.

### 3.6. Evaluation of Drug-Likeness

**Figure 14:**
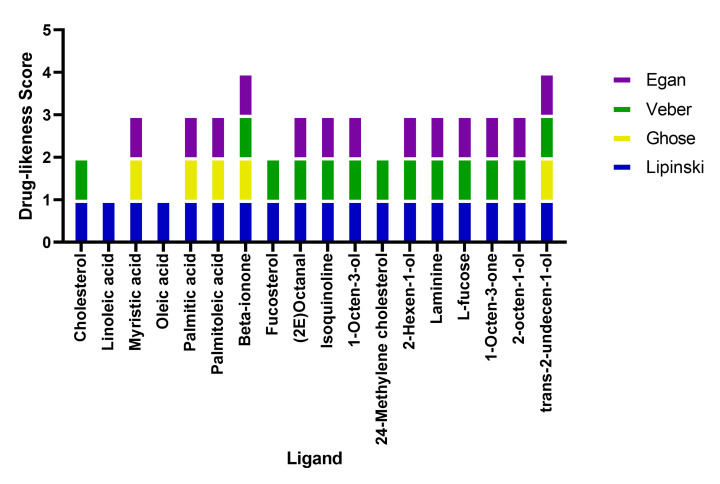
Drug-likeness evaluation of *S. japonica*-derived ligands based on five different criteria from SwissADME: Lipinski, Ghose, Veber, Muegge, and Egan. Each bar represents the cumulative score of each ligand across the five rule-based filters.

Drug-likeness was evaluated for 18 *S. japonica* compounds using SwissADME and five rule-based filters (Lipinski, Ghose, Veber, Egan, Muegge) [33,34,35,36,37]. Thirteen compounds satisfied at least three filters, with trans-2-undecen-1-ol meeting four criteria except Muegge, indicating good drug-likeness (Figure 14). Most compounds met Lipinski and Egan filters, suggesting favorable oral absorption and membrane permeability, though none met Muegge criteria.

Fucosterol and 24-methylene cholesterol satisfied molecular weight, TPSA, and rotatable bond thresholds but exceeded the log P limit (>5) and possessed only one heteroatom. These features indicate excessive lipophilicity and low polarity, implying limited solubility and restricted systemic distribution—common challenges for sterol-based bioactives.

### 3.7. ADMET Profile

The ADMET properties of 18 ligands were predicted using pkCSM (Table 2, Table 3, Table 4, Table 5 and Table 6). Most compounds exhibited high intestinal absorption (>90%), particularly 1-octen-3-one (96.77%), (2E)-octenal (96.06%), and 24-methylene cholesterol (95.88%) (Table 2). All ligands except L-fucose demonstrated strong Caco-2 permeability (log Papp > 0.9), with palmitoleic acid showing the highest value (1.565). Cholesterol, fucosterol, and 24-methylene cholesterol were predicted as P-glycoprotein (P-gp) inhibitors, whereas linoleic acid, oleic acid, and isoquinoline were P-gp substrates [38].

Regarding distribution, nine compounds had log BB > 0.3, suggesting potential blood–brain barrier (BBB) penetration [39], and linoleic and oleic acids exhibited VDss > 0.45, indicating good systemic distribution (Table 3) [40]. For metabolism, cholesterol, fucosterol, and palmitic acid were predicted as CYP3A4 substrates, while oleic acid and laminine were CYP2D6 substrates (Table 4). Linoleic and oleic acids were also identified as CYP2C19 inhibitors, suggesting possible drug–drug interactions [41].

Excretion profiles indicated total clearance between log 0.38 and 2.06, with linoleic and oleic acids showing the highest rates (Table 5). All compounds were non-substrates of OCT2, implying renal safety [42]. Toxicity predictions revealed all compounds to be non-mutagenic and non-hepatotoxic, with LD_50_, LOAEL, and MTD values within acceptable ranges (Table 6). However, cholesterol, linoleic acid, fucosterol, and 1-octen-3-ol were flagged as potential hERG II inhibitors, suggesting cardiotoxic risk through QT prolongation [43].

## 4. Conclusions

Overall, this study suggests that *S. japonica* ethanol extracts possess antioxidant potential and may exhibit selective antibacterial activity, particularly against *S. aureus.* Among the tested extracts, the 60% ethanol extracts showed relatively strong antioxidant activity, whereas the antibacterial effects were species dependent. Molecular docking and ADMET analyses indicated that several *S. japonica*-derived compounds, including sterol derivatives such as fucosterol and 24-methylene cholesterol, may contribute to the observed bioactivities and could serve as candidate compounds for further investigation. However, the antibacterial effects observed in this study should be interpreted cautiously, as complete bactericidal activity was not achieved and several predicted pharmacokinetic and toxicity-related limitations were identified. These findings suggest that *S. japonica* may serve as a potential source of bioactive compounds for antioxidant and antimicrobial-related applications, providing supporting experimental and computational evidence.

## Figures and Tables

**Table 1:** Minimum inhibitory concentration (MIC) of *S. japonica* ethanol extracts against *E. coli* and *S. aureus*.

Bacterial Strain	Sample	MIC (mg/mL)		
		30% EtOH	60% EtOH	90% EtOH
*E. coli*	GLJE ^a^	10	10	10
	WLJE ^b^	10	10	10
	Gentamicin	0.005		
	Control (EtOH)	10	–	–
*S. aureus*	GLJE	10	2.5	5
	WLJE	10	1.25	5
	Gentamicin	0.005		
	Control (EtOH)	10	–	–

^a^ GLJE: *S. japonica* ethanol extract from Gijang, ^b^ WLJE: *S. japonica* ethanol extract from Wando.

**Table 2:** Absorption predictions of *S. japonica*-derived compounds using pkCSM.

Ligands	Water Solubility (log mol/L)	Caco-2 Permeability (logPapp in 10^−6^ cm/s)	Intestinal Absorption (Human) (% Absorbed)	P-Glycoprotein Substrate	P-Glycoprotein I/II Inhibitor
Cholesterol	−7.042	1.275	94.929	No	Yes
Linoleic acid	−5.554	1.284	92.227	Yes	No
Myristic acid	−4.952	1.56	92.691	No	No
Oleic acid	−5.625	1.277	91.721	Yes	No
Palmitic acid	−5.562	1.558	92.004	No	No
Palmitoleic acid	−5.477	1.565	92.51	No	No
β-ionone	−3.552	1.186	95.797	No	No
Fucosterol	−6.917	1.279	96.061	No	Yes
(2E)-octenal	−2.84	1.493	95.588	No	No
Isoquinoline	−2.358	1.356	96.924	Yes	No
1-octen-3-ol	−1.34	1.089	94.241	No	No
24-methylene cholesterol	−6.934	1.273	95.884	No	Yes
2-hexen-1-ol	−1.082	1.48	93.765	No	No
Laminine	−0.763	0.964	87.965	No	No
L-fucose	0.252	0.222	61.19	No	No
1-octen-3-one	−2.131	1.107	96.766	No	No
2-octen-1-ol	−2.336	1.478	93.059	No	No
Trans-2-undecen-1-ol	−4.227	1.475	92.028	No	No

**Table 3:** Distribution predictions of *S. japonica*-derived compounds using pkCSM.

Ligands	VDss (Human) (log L/kg)	BBB Permeability (log BB)	CNS Permeability (log PS)
Cholesterol	0.299	0.794	−1.374
Linoleic acid	0.594	−0.005	−3.961
Myristic acid	−0.578	−0.027	−1.925
Oleic acid	0.626	−0.032	−4.014
Palmitic acid	−0.543	−0.111	−1.816
Palmitoleic acid	−0.574	−0.084	−1.763
β-ionone	0.252	0.595	−2.333
Fucosterol	0.104	0.807	−1.326
(2E)-octenal	0.147	0.658	−1.948
Isoquinoline	0.397	0.26	−2.961
1-octen-3-ol	−0.008	0.493	−2.034
24-methylene cholesterol	0.195	0.805	−1.421
2-hexen-1-ol	0.024	0.16	−2.25
Laminine	−0.051	−0.201	−3.031
L-fucose	−0.095	−0.909	−4.979
1-octen-3-one	0.063	0.655	−2.153
2-octen-1-ol	0.151	0.526	−2.121
Trans-2-undecen-1-ol	0.316	0.694	−1.957

**Table 4:** Metabolism predictions of *S. japonica*-derived compounds using pkCSM.

Ligands	CYP2D6 Substrate	CYP3A4 Substrate	CYP1A2 Inhibitor	CYP2C19 Inhibitor	CYP2C9 Inhibitor	CYP2D6 Inhibitor	CYP3A4 Inhibitor
Cholesterol	No	Yes	No	No	No	No	No
Linoleic acid	No	No	No	Yes	No	No	No
Myristic acid	No	No	No	No	No	No	No
Oleic acid	Yes	No	No	Yes	No	No	No
Palmitic acid	No	Yes	No	No	No	No	No
Palmitoleic acid	No	Yes	No	No	No	No	No
β-ionone	No	No	No	No	No	No	No
Fucosterol	No	Yes	No	No	No	No	No
(2E)-octenal	No	No	No	No	No	No	No
Isoquinoline	No	No	No	No	No	No	No
1-octen-3-ol	No	No	No	No	No	No	No
24-methylene cholesterol	No	Yes	No	No	No	No	No
2-hexen-1-ol	No	No	No	No	No	No	No
Laminine	Yes	No	No	No	No	No	No
L-fucose	No	No	No	No	No	No	No
1-octen-3-one	No	No	No	No	No	No	No
2-octen-1-ol	No	No	No	No	No	No	No
Trans-2-undecen-1-ol	No	No	No	No	No	No	No

**Table 5:** Excretion predictions of *S. japonica*-derived compounds using pkCSM.

Ligands	Total Clearance (log mL/min/lg)	Renal OCT2 Substrate
Cholesterol	0.589	No
Linoleic acid	2.058	No
Myristic acid	1.693	No
Oleic acid	2.007	No
Palmitic acid	1.763	No
Palmitoleic acid	1.817	No
β-ionone	1.315	No
Fucosterol	0.619	No
(2E)-octenal	0.37	No
Isoquinoline	0.377	No
1-octen-3-ol	0.461	No
24-methylene cholesterol	0.604	No
2-hexen-1-ol	0.382	No
Laminine	0.594	No
L-fucose	0.585	No
1-octen-3-one	0.442	No
2-octen-1-ol	0.431	No
Trans-2-undecen-1-ol	1.745	No

**Table 6:** Toxicity predictions of *S. japonica*-derived compounds using pkCSM.

Ligands	AMES Toxicity	MAX. Tolerated Dose (Human) (log mg/kg/day)	hERG I Inhibitor	hERG II Inhibitor	Oral Rat Acute Toxicity (LD_50_) (mol/kg)	Oral Rat Chronic Toxicity (LOAEL) (log mg/kg_bw/day)	Hepato- toxicity
Cholesterol	No	−0.362	No	Yes	2.366	1.192	No
Linoleic acid	No	0.192	No	Yes	2.513	0.312	No
Myristic acid	No	−0.559	No	No	1.477	3.034	No
Oleic acid	No	0.204	No	No	1.417	3.259	No
Palmitic acid	No	−0.708	No	No	1.44	3.181	No
Palmitoleic acid	No	−0.713	No	No	1.449	3.109	No
β-ionone	No	0.581	No	No	1.855	1.217	No
Fucosterol	No	−0.374	No	Yes	2.847	1.119	No
(2E)-octenal	No	0.63	No	No	1.822	2.032	No
Isoquinoline	No	0.663	No	No	2.23	1.92	No
1-octen-3-ol	No	0.792	No	Yes	2.75	1.125	No
24-methylene cholesterol	No	−0.358	No	No	1.703	1.999	No
2-hexen-1-ol	No	0.997	No	No	1.735	1.767	No
Laminine	No	1.015	No	No	1.432	0.501	No
L-fucose	No	2.193	No	No	1.331	3.698	No
1-octen-3-one	No	0.869	No	No	1.828	1.951	No
2-octen-1-ol	No	0.74	No	No	1.639	1.929	No
Trans-2-undecen-1-ol	No	0.387	No	No	1.552	2.183	No

## Data Availability

The data analyzed in the present study are available within the article and Supplementary Materials.

## Supplementary Materials

The following supporting information can be downloaded at: https://www.mdpi.com/article/10.3390/pathogens15060576/s1, Table S1: Chemical structure of the compounds in *S. japonica* used for molecular docking study. Table S2: Grid box parameters and redocking validation results for molecular docking.

## References

[1] Bushra R., Saeed F., Ahmed Z., Naeem S., Ishaq J., Saleem S.Y. (2025). Curbing Anti-Microbial Resistance of Synthetic Medicinal Agents Using Herbal Drug Alternatives: Current Trends and Future Insights. Prospect. Pharm. Sci..

[2] Oluyele O. (2025). Antimicrobial Efficacy and Time-Kill Kinetics of *Phoenix dactylifera* L. Seed Oil Against Multidrug Resistant Pathogens from Cancer Patients. Prospect. Pharm. Sci..

[3] Lomartire S., Gonçalves A.M.M. (2022). An Overview of Potential Seaweed-Derived Bioactive Compounds for Pharmaceutical Applications. Mar. Drugs.

[4] Lu W.-J., Lin H.-J., Hsu P.-H., Lai M., Chiu J.-Y., Lin H.-T.V. (2019). Brown and Red Seaweeds Serve as Potential Efflux Pump Inhibitors for Drug-Resistant *Escherichia coli*. Evid. Based. Complement. Altern. Med..

[5] Wan H., Zhang Y.-X., Gao Z.-C., Shan G.-Y., Liu F., Li H.-J. (2025). Exploring the Potential of Fucoidan from *Laminaria japonica*: A Comprehensive Review of Its Biological Activities and Benefits for Human. Int. J. Biol. Macromol..

[6] Liu X. (2020). Extraction and Anti-Bacterial Effects of Edible Brown Algae Extracts.

[7] Generalić Mekinić I., Skroza D., Šimat V., Hamed I., Čagalj M., Popović Perković Z. (2019). Phenolic Content of Brown Algae (Pheophyceae) Species: Extraction, Identification, and Quantification. Biomolecules.

[8] Patra J.K., Das G., Baek K.-H. (2015). Chemical Composition and Antioxidant and Antibacterial Activities of an Essential Oil Extracted from an Edible Seaweed, *Laminaria japonica* L.. Molecules.

[9] Cai J., Feng J., Xie S., Wang F., Xu Q. (2014). *Laminaria japonica* Extract, an Inhibitor of *Clavibater michiganense* subsp. Sepedonicum. PLoS ONE.

[10] Kanazawa K., Ozaki Y., Hashimoto T., Das S.K., Matsushita S., Hirano M., Okada T., Komoto A., Mori N., Nakatsuka M. (2008). Commercial-Scale Preparation of Biofunctional Fucoxanthin from Waste Parts of Brown Sea Algae *Laminalia japonica*. Food Sci. Technol. Res..

[11] Wang J., Zhang Q., Zhang Z., Song H., Li P. (2010). Potential Antioxidant and Anticoagulant Capacity of Low Molecular Weight Fucoidan Fractions Extracted from *Laminaria japonica*. Int. J. Biol. Macromol..

[12] Castejón N., Parailloux M., Izdebska A., Lobinski R., Fernandes S.C.M. (2021). Valorization of the Red Algae Gelidium Sesquipedale by Extracting a Broad Spectrum of Minor Compounds Using Green Approaches. Mar. Drugs.

[13] (2023). Performance Standards for Antimicrobial Disk and Dilution Susceptibility Tests for Bacteria Isolated from Animals.

[14] Selvaraj J., Vishnu Priya V., Vijayalakshmi P., Ponnulakshmi R. (2021). In Silico and in Vitro Study on the Inhibition of FtsZ Protein of *Staphylococcus aureus* by Active Compounds from *Andrographis paniculata*. J. Biol. Act. Prod. Nat..

[15] Dallakyan S., Olson A.J. (2015). Small-Molecule Library Screening by Docking with PyRx. Methods Mol. Biol..

[16] Teodosio J.J.R., Dizon K.A.H., Bruna J.R., Sollesta J.V.N., Villorente Z.M., Saludes J.P., Dalisay D.S. (2026). Biochanin A, a Plant Isoflavone, Disrupts Peptidoglycan Biosynthesis by Downregulating FemA and FemB, and Impairs Cell Wall Integrity in Multidrug-Resistant Staphylococcus Aureus. Antibiotics.

[17] McCormick J.K., Tripp T.J., Llera A.S., Sundberg E.J., Dinges M.M., Mariuzza R.A., Schlievert P.M. (2003). Functional Analysis of the TCR Binding Domain of Toxic Shock Syndrome Toxin-1 Predicts Further Diversity in MHC Class II/Superantigen/TCR Ternary Complexes. J. Immunol..

[18] Kahlon A.K., Roy S., Sharma A. (2010). Molecular Docking Studies to Map the Binding Site of Squalene Synthase Inhibitors on Dehydrosqualene Synthase of Staphylococcus Aureus. J. Biomol. Struct. Dyn..

[19] Daina A., Michielin O., Zoete V. (2017). SwissADME: A Free Web Tool to Evaluate Pharmacokinetics, Drug-Likeness and Medicinal Chemistry Friendliness of Small Molecules. Sci. Rep..

[20] Pires D.E.V., Blundell T.L., Ascher D.B. (2015). PkCSM: Predicting Small-Molecule Pharmacokinetic and Toxicity Properties Using Graph-Based Signatures. J. Med. Chem..

[21] Jang E.J., Kim S.C., Lee J.-H., Lee J.R., Kim I.K., Baek S.Y., Kim Y.W. (2018). Fucoxanthin, the Constituent of *Laminaria japonica*, Triggers AMPK-Mediated Cytoprotection and Autophagy in Hepatocytes under Oxidative Stress. BMC Complement. Altern. Med..

[22] Karpiński T.M., Adamczak A. (2019). Fucoxanthin—An Antibacterial Carotenoid. Antioxidants.

[23] Singleton V.L., Orthofer R., Lamuela-Raventós R.M. (1999). [14] Analysis of Total Phenols and Other Oxidation Substrates and Antioxidants by Means of Folin-Ciocalteu Reagent. Methods Enzymol..

[24] Fu C.W.F., Ho C.W., Yong W.T.L., Abas F., Tan T.B., Tan C.P. (2016). Extraction of Phenolic Antioxidants from Four Selected Seaweeds Obtained from Sabah. Int. Food Res. J..

[25] Gomes L., Monteiro P., Cotas J., Gonçalves A.M.M., Fernandes C., Gonçalves T., Pereira L. (2022). Seaweeds’ Pigments and Phenolic Compounds with Antimicrobial Potential. Biomol. Concepts.

[26] Saxena D., Maitra R., Bormon R., Czekanska M., Meiers J., Titz A., Verma S., Chopra S. (2023). Tackling the Outer Membrane: Facilitating Compound Entry into Gram-Negative Bacterial Pathogens. npj Antimicrob. Resist..

[27] Zgurskaya H.I., Rybenkov V.V. (2019). Permeability Barriers of Gram-negative Pathogens. Ann. N. Y. Acad. Sci..

[28] Čmiková N., Galovičová L., Miškeje M., Borotová P., Kluz M., Kačániová M. (2022). Determination of Antioxidant, Antimicrobial Activity, Heavy Metals and Elements Content of Seaweed Extracts. Plants.

[29] da Silva F.E.F., Ávila F.d.N., Pereira N.M.O., de Freitas M.D., Pessoa O.D.L., da Fonseca A.M., da Costa J.G.M., Santiago G.M.P. (2023). Semisynthesis, in Silico Study and in Vitro Antibacterial Evaluation of Fucosterol Derivatives. Steroids.

[30] Touhtouh J., Laghmari M., Benali T., Aanniz T., Akhazzane M., Goh K.W., Al Abdulmonem W., Bouyahya A., Zengin G., Hammani K. (2024). Evaluation of Antioxidant, Antimicrobial, Antidiabetic, Anti-Tyrosinase, and Neuroprotective Effects of β-Ionone: In Vitro and in Silico Analysis. Results Chem..

[31] Kubiak J., Szyk P., Czarczynska-Goslinska B., Goslinski T. (2025). Flavonoids, Chalcones, and Their Fluorinated Derivatives—Recent Advances in Synthesis and Potential Medical Applications. Molecules.

[32] Govender N., Zulkifli N.S., Badrul Hisham N.F., Ab Ghani N.S., Mohamed-Hussein Z.-A. (2022). Pea Eggplant (*Solanum torvum* Swartz) Is a Source of Plant Food Polyphenols with SARS-CoV Inhibiting Potential. PeerJ.

[33] Egan W.J., Merz Kenneth M., Baldwin J.J. (2000). Prediction of Drug Absorption Using Multivariate Statistics. J. Med. Chem..

[34] Ghose A.K., Viswanadhan V.N., Wendoloski J.J. (1998). A Knowledge-Based Approach in Designing Combinatorial or Medicinal Chemistry Libraries for Drug Discovery. 1. A Qualitative and Quantitative Characterization of Known Drug Databases. J. Comb. Chem..

[35] Lipinski C.A., Lombardo F., Dominy B.W., Feeney P.J. (2012). Experimental and Computational Approaches to Estimate Solubility and Permeability in Drug Discovery and Development Settings. Adv. Drug Deliv. Rev..

[36] Muegge I., Heald S.L., Brittelli D. (2001). Simple Selection Criteria for Drug-like Chemical Matter. J. Med. Chem..

[37] Veber D.F., Johnson S.R., Cheng H.-Y., Smith B.R., Ward K.W., Kopple K.D. (2002). Molecular Properties That Influence the Oral Bioavailability of Drug Candidates. J. Med. Chem..

[38] Fromm M.F. (2003). Importance of P-glycoprotein for Drug Disposition in Humans. Eur. J. Clin. Investig..

[39] Matondo A., Kilembe J.T., Ngoyi E.M., Kabengele C.N., Kasiama G.N., Lengbiye E.M., Mbadiko C.M., Inkoto C.L., Bongo G.N., Gbolo B.Z. (2021). Oleanolic Acid, Ursolic Acid and Apigenin from Ocimum Basilicum as Potential Inhibitors of the SARS-CoV-2 Main Protease: A Molecular Docking Study. Int. J. Pathog. Res..

[40] Mvondo J.G.M., Matondo A., Mawete D.T., Bambi S.-M.N., Mbala B.M., Lohohola P.O. (2021). In Silico ADME/T Properties of Quinine Derivatives Using SwissADME and PkCSM Webservers. Int. J. Trop. Dis. Health.

[41] Yeni Y., Rachmania R.A. (2022). The Prediction of Pharmacokinetic Properties of Compounds in *Hemigraphis alternata* (Burm.F.) T. Ander Leaves Using PkCSM. Indones. J. Chem..

[42] Saad A.A.A., Zhang F., Mohammed E.A.H., Wu X. (2022). Clinical Aspects of Drug–Drug Interaction and Drug Nephrotoxicity at Renal Organic Cation Transporters 2 (OCT2) and Multidrug and Toxin Exclusion 1, and 2-K (MATE1/MATE2-K). Biol. Pharm. Bull..

[43] Yuan Y., Bai X., Luo C., Wang K., Zhang H. (2015). The Virtual Heart as a Platform for Screening Drug Cardiotoxicity. Br. J. Pharmacol..

