# Supplementary material for: Marine-Derived Sterols from Saccharina japonica: Potential Antibacterial Activity and Target Prediction Against Bacterial Pathogens Through Integrated In Vitro and In Silico Approaches
Source: Pathogens. 2026 May 27;15(6):576. doi: 10.3390/pathogens15060576 (PMC13304927; doi:10.3390/pathogens15060576)
Supplement: Supplementary file 1 [file pathogens-15-00576-s001.zip › pathogens-4285612-supplementary.pdf]

Table S1. Chemical structure of the compounds in *S. japonica* used for molecular docking study

No	Name of the compound	IUPAC name	CID	Formula	Structure
1	Cholesterol	(3S,8S,9S,10R,13R,14S,17R)-10,13-dimethyl-17-[(2R)-6-methylheptan-2-yl]-2,3,4,7,8,9,11,12,14,15,16,17-dodecahydro-1H-cyclopenta[a]phenanthren-3-ol	5997	$C_{27}H_{46}O$	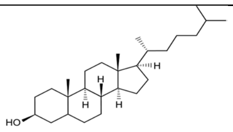
2	Linoleic acid	(9Z,12Z)-octadeca-9,12-dienoic acid	5280450	$C_{18}H_{32}O_2$	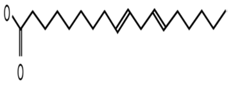
3	Myristic acid	tetradecanoic acid	11005	$C_{14}H_{28}O_2$	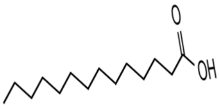
4	Oleic acid	(Z)-octadec-9-enoic acid	445639	$C_{18}H_{34}O_2$	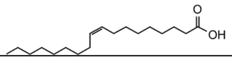
5	Palmitic acid	hexadecanoic acid	985	$C_{16}H_{32}O_2$	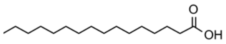
6	Palmitoleic acid	(Z)-hexadec-9-enoic acid	445638	$C_{16}H_{30}O_2$	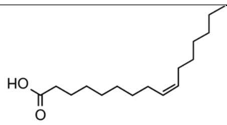
7	β-ionone	(E)-4-(2,6,6-trimethylcyclohexen-1-yl)but-3-en-2-one	638014	$C_{13}H_{20}O$	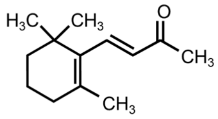
8	Fucosterol	(3S,8S,9S,10R,13R,14S,17R)-10,13-dimethyl-17-[(E,2R)-5-propan-2-ylhept-5-en-2-yl]-2,3,4,7,8,9,11,12,14,15,16,17-dodecahydro-1H-cyclopenta[a]phenanthren-3-ol	5281328	$C_{29}H_{48}O$	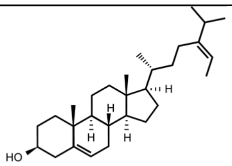
9	(2E)-Octenal	(E)-oct-2-enal	5283324	$C_8H_{14}O$	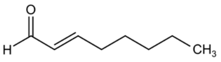
10	Isoquinoline	isoquinoline	8405	C_9H_7N	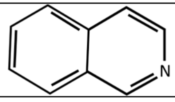
11	1-Octen-3-ol	oct-1-en-3-ol	18827	$C_8H_{16}O$	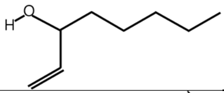
12	24-Methylene cholesterol	[(3S,8S,9S,10R,13R,14S,17R)-10,13-dimethyl-17-[(2R)-6-methyl-5-methylideneheptan-2-yl]-2,3,4,7,8,9,11,12,14,15,16,17-dodecahydro-1H-cyclopenta[a]phenanthren-3-yl] hydrogen sulfate	24779661	$C_{28}H_{46}O_4S$	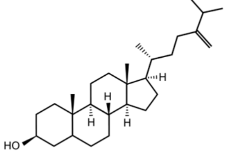

13	2-Hexen-1-ol	(E)-hex-2-en-1-ol	5318042	$C_6H_{12}O$	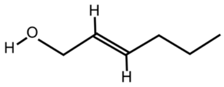
14	Laminine	2-amino-6-(trimethylazaniumyl)hexanoate	159659	$C_9H_{20}N_2O_2$	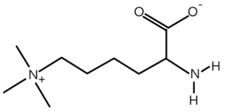
15	L-fucose	(3S,4R,5S,6S)-6-methyloxane-2,3,4,5-tetrol	17106	$C_6H_{12}O_5$	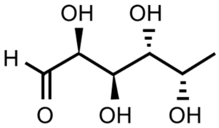
16	1-Octen-3-one	oct-1-en-3-one	61346	$C_8H_{14}O$	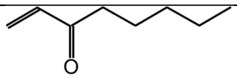
17	2-octen-1-ol	(E)-oct-2-en-1-ol	5318599	$C_8H_{16}O$	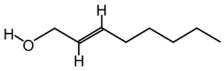
18	Trans-2-undecen-1-ol	(E)-undec-2-en-1-ol	5365004	$C_{11}H_{22}O$	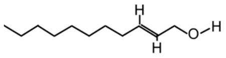

Table S2. Grid box parameters and redocking validation results for molecular docking

PDB ID	Docking site	Grid center coordinates (X, Y, Z)	Grid box (Å)	RMSD (Å)
1KZN	CBN (clorobiocin)	19.19, 30.45, 34.85	22 × 22 × 22	0.63
1UAE	FFQ, UD1	43.73, 21.65, 40.93	22 × 22 × 22	0.42
3FV5	1EU	9.86, −1.34, 4.84	22 × 22 × 22	1.10
5L3J	6G9	−13.30, 18.21, 21.34	22 × 22 × 22	1.99
1JIJ	SB-239629	−11.48, 15.09, 85.95	22 × 22 × 22	1.35
1LRZ	ASP150, GLY330, PHE363, LYS383	42.22, 53.09, 95.00	22 × 22 × 22	-
2QIL	GLY16, TRP116, GLU132, HIS135, GLN136, GLN139	35.95, 51.99, 23.34	22 × 22 × 22	-
2ZCO	HIS18, ARG45, ASP48, ASP52, TYR129, GLN165, ASN168, ASP172	53.63, 6.39, 50.68	22 × 22 × 22	-
3TTZ	07N	0.41, 3.18, 23.92	22 × 22 × 22	1.35
